# Serological signature of tick-borne pathogens in Scandinavian brown bears over two decades

**DOI:** 10.1186/s13071-015-0967-2

**Published:** 2015-07-28

**Authors:** Lye Paillard, Krista L. Jones, Alina L. Evans, Jérémy Berret, Maxime Jacquet, Reto Lienhard, Mahmoud Bouzelboudjen, Jon M. Arnemo, Jon E. Swenson, Maarten J. Voordouw

**Affiliations:** Laboratory of Ecology and Evolution of Parasites, Institute of Biology, University of Neuchâtel, Rue Emile-Argand 11, 2000 Neuchâtel, Switzerland; Department of Forestry and Wildlife Management, Faculty of Forestry and Wildlife Management, Hedmark University College, Campus Evenstad, NO-2418, Elverum, Norway; ADMED Microbiologie, Boucle de Cydalise 16, 2300 la Chaux-de-Fonds, Switzerland; Informatics and Telematics Service, University of Neuchâtel, Rue Emile-Argand 11, 2000 Neuchâtel, Switzerland; Department of Wildlife, Fish and Environmental Studies, Faculty of Forest Sciences, Swedish University of Agricultural Sciences, SE-901 83 Umeå, Sweden; Department of Ecology and Natural Resources Management, Norwegian University of Life Sciences, Postbox 5003, NO-1432 Ås, Norway; Norwegian Institute for Nature Research, NO-7485 Trondheim, Norway

**Keywords:** *Borrelia burgdorferi*, *Ixodes ricinus*, Lyme disease, Scandinavia, Serology, Tick-borne diseases, Tick-borne encephalitis virus, *Ursus arctos*, Vector-borne diseases, Zoonoses

## Abstract

**Background:**

Anthropogenic disturbances are changing the geographic distribution of ticks and tick-borne diseases. Over the last few decades, the tick *Ixodes ricinus* has expanded its range and abundance considerably in northern Europe. Concurrently, the incidence of tick-borne diseases, such as Lyme borreliosis and tick-borne encephalitis, has increased in the human populations of the Scandinavian countries.

**Methods:**

Wildlife populations can serve as sentinels for changes in the distribution of tick-borne diseases. We used serum samples from a long-term study on the Scandinavian brown bear, *Ursus arctos*, and standard immunological methods to test whether exposure to *Borrelia burgdorferi* sensu lato, the causative agent of Lyme borreliosis, and tick-borne encephalitis virus (TBEV) had increased over time. Bears had been sampled over a period of 18 years (1995–2012) from a southern area, where *Ixodes ricinus* ticks are present, and a northern area where ticks are uncommon or absent.

**Results:**

Bears had high levels of IgG antibodies against *B. burgdorferi* sensu lato but not TBEV. Bears at the southern area had higher values of anti-*Borrelia* IgG antibodies than bears at the northern area. Over the duration of the study, the value of anti-*Borrelia* IgG antibodies increased in the southern area but not the northern area. Anti-*Borrelia* IgG antibodies increased with the age of the bear but declined in the oldest age classes.

**Conclusions:**

Our study is consistent with the view that ticks and tick-borne pathogens are expanding their abundance and prevalence in Scandinavia. Long-term serological monitoring of large mammals can provide insight into how anthropogenic disturbances are changing the distribution of ticks and tick-borne diseases.

## Background

The incidence of tick-borne diseases, such as Lyme borreliosis (LB) and tick-borne encephalitis (TBE), has increased over the last few decades in a number of European countries [1–3]. One explanation for the increase in tick-borne infections is that the distributional area and abundance of the principal vector, *Ixodes ricinus,* have increased [4–8]. Consistent with this explanation, studies in Norway and Sweden have shown that the abundance and prevalence of ticks and tick-borne diseases have increased during this time [9, 10]. The distribution of ticks and tick-borne diseases has expanded northward, to higher altitudes, and to new inland regions [9]. In addition, ticks have increased in abundance where they were already present in central and south Sweden [10, 11]. Studies in other parts of the world also have reported changes in the distribution of ticks and tick-borne diseases [12–15].

Climate change could drive changes in the distribution of ticks because these arthropods are very sensitive to temperature and humidity [4, 7]. In southern Sweden, the increase in the incidence of LB was positively correlated with a rise in monthly mean temperature [16]. Climate change could also influence the distribution and abundance of ticks and tick-borne diseases via indirect effects on vegetation [15, 17] and important reservoir hosts like rodents [1]. The climate change hypothesis for the emergence of tick-borne diseases in Europe is controversial [2, 7, 18, 19]. Alternative anthropogenic explanations include changes in agriculture and land use that have increased the amount of suitable tick habitat [3, 19]. Additional explanations include improved reporting, diagnosis, and awareness of tick-borne diseases [9, 20], changes in human behaviour that increase contact with ticks [7, 16, 19], and even the socio-political changes in Eastern Europe following the collapse of communism [3, 21].

Immunological methods are widely used to determine whether vertebrate populations have been exposed to tick-borne pathogens [22–24]. The study of changes in the IgG antibody response over time can provide insight into the temporal dynamics of tick-borne diseases [25]. The purpose of our study was to test whether the observed increase in the incidence of tick-borne diseases over the last two decades in Scandinavia could be detected in wild animal sera.

To address this question, we used standard immunological methods to quantify the IgG antibody response against two common tick-borne pathogens in the brown bear (*Ursus arctos*). Long-lived mammals, such as brown bears, can be repeatedly exposed to ticks and are therefore expected to amplify the immunological signature of tick-borne pathogens. Previous studies in Europe and North America have shown that bears can be used as sentinels for tick-borne diseases [26–29]. We investigated the strength of the IgG antibody response against the spirochete bacterium *Borrelia burgdorferi* sensu lato (s. l.), the causative agent of Lyme borreliosis, and the tick-borne encephalitis virus (TBEV). We chose these two tick-borne pathogens because they are present in Scandinavia [2, 17, 18, 30–34] and because reliable ELISA tests are commercially available [35–37].

The brown bears were captured at a southern and a northern area in Sweden over a period of 18 years (1995 to 2012). In the southern area, populations of *I. ricinus* have increased substantially from the early 1990s to 2008 [10]. In the northern area, by contrast, there have been much fewer reports of *I. ricinus* as of 2008 [10]. We therefore predicted that the immune response against tick-borne pathogens would be much stronger in bears from the southern area than bears from the northern area. We also predicted that the immune response against tick-borne pathogens in bear sera would increase over the 18 years of the study in the southern area but not the northern area.

## Methods

### Collection of bear serum samples

**Fig. 1:**
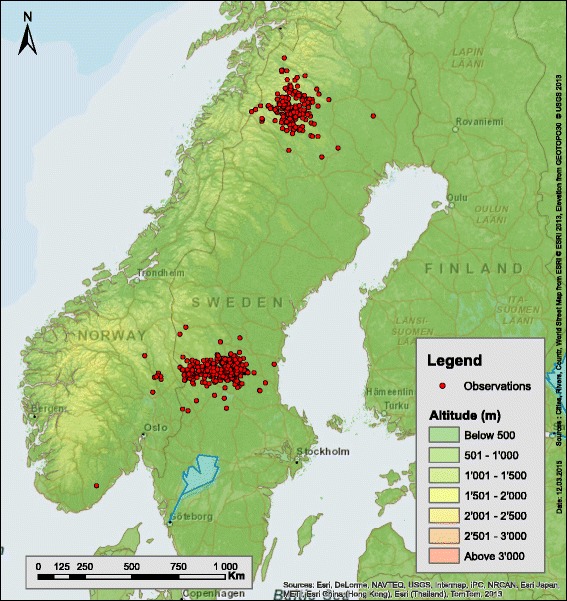
Map showing the study area in Sweden. Map showing the geographic location of the northern and southern area in Scandinavia where the wild brown bears were captured. Each bear serum sample is represented by one dot

The serum samples were obtained from a long-term study of the brown bear in Sweden. These samples spanned 18 years (1995 to 2012) and came from two distinct regions that are approximately 600 km apart. The southern area was centred in Dalarna and Gävleborg counties in central Sweden (61°30′0″N, 17°0′0″E), with a rolling landscape of coniferous forest dominated by commercial plantations of Scots pine (*Pinus sylvestris*) and Norway spruce (*Picea abies*). The northern area, centred in Norrbotten County (66°36′23″N, 19°49′23″E), is mountainous, with altitudes up to 2000 m and is covered by coniferous forest of Scots pine and Norway spruce at lower altitudes and subalpine forests dominated by birch (*Betula pubescens*) and willows (*Salix* spp.) at higher altitudes (Fig. 1). Details of how the bears were captured have been described elsewhere [38]. Briefly, bears were immobilized by darting from helicopter in the early spring upon emergence from their winter dens. We determined the sex and age of the individuals, collected blood samples, and gave them a unique identification marking. We combined our data into five age groups: yearlings (0–1 years), juveniles (2–3 years), young adults (4–9 years), adults (10–14 years), and old adults (15–29 years). Our data set contained 1,172 serum samples collected from 569 individual bears (mean = 2.06 samples/bear; range = 1 to 9 samples/bear). The bear serum samples were kept at −20 °C until further analysis.

### Detection of antibodies against *Borrelia* pathogens

We used an ELISA assay (*Borrelia* microplate IgG) to test whether the bears had developed specific antibodies against *Borrelia* pathogens. The 1172 bear serum samples were randomly assigned to one of 14 96-well commercial ELISA plates (Virion; SERION ELISA). Each ELISA plate contained 84 wild bear serum samples, four positive controls (sera from laboratory mice experimentally infected with *B. afzelii*), four negative controls (sera from uninfected laboratory mice), and four bear serum samples from zoological parks. The serum samples from the zoo bears and the positive and negative controls functioned as a quality control of the ELISA assay. The laboratory mice (positive controls) had been infected with *B. afzelii* by infesting them with *B. afzelii*-infected *I. ricinus* nymphs (unpublished data). To determine the repeatability of the ELISA assay, we also repeated the assay for one randomly selected plate.

### Ethical approval

The Ethical Committee on Animal Experiments, Uppsala, Sweden (# C 7/12) and the National Animal Research Authority, Oslo, Norway (# 2013/33387) approved the sampling of blood from captured bears. All experiments involving mice respected the Swiss legislation on animal experimentation and were authorized by the Veterinary Service of the Canton of Neuchâtel (Authorization number NE2/2012).

Most serological studies on bears and other wild animals use serum dilutions of 1:50 to 1:200 [39–44]. Following the recommendations of the manufacturer, we used a serum dilution of 1:100 by adding 10 μl of serum to 990 μl of 1× PBS. We incubated the plates with 100 μl of the diluted sera for 45 min. We removed the sera and washed the wells three times with 200 μl of washing solution (1× PBS with 0.1 % Tween). We used Protein A conjugated to horseradish peroxidase (INVITROGEN, Thermo scientific) as the secondary antibody because it has been shown to bind the IgG antibodies of a variety of mammals [45]. We confirmed independently that Protein A was capable of binding to IgG antibodies of bears and mice. We added 100 μl of the secondary antibody diluted 1:5000 in 1× PBS and incubated the plates at room temperature for 45 min. We again washed the plates three times with washing solution. We added 100 μl of TMB solution (Thermo scientific) to each well to produce a colour reaction. The absorbance was read at a wavelength of 652 nm every 2 min for 1 h with a plate reader (BIO-TEK Instruments, program KC4™ v3.2).

### Detection of antibodies against TBEV

We used an ELISA assay (FSME (TBE) Microtiter plates IgG) to test whether the bears had developed specific antibodies against TBEV. The ELISA protocol was the same as described for *Borrelia*. For the positive and negative controls, we used goat serum samples from a previous study [46] in addition to the bear serum samples from the zoological park. The seropositive status of these goat serum samples had been determined using a serum neutralization test, which is considered the gold standard in the diagnosis of TBEV. We found that Protein G was much more effective than Protein A at binding goat IgG. For the TBEV ELISA assay, we therefore used two different secondary antibodies: Protein A for the bear samples and Protein G for the goat samples.

### Collection of tissue samples from bears

We used quantitative PCR (qPCR) to test bear tissue samples for infection with *Borrelia* pathogens. We collected tissue samples from 16 bears that had been killed legally by private hunters on different days during the last three weeks of the month of August 2014 in the southern area. No bears were killed for the purpose of this study. For each bear, all tissue samples were collected within a few hours following death from the following organs: skin, liver, kidney, bladder, and arteries. One tissue sample was unusable, resulting in a total of 79 tissue samples. Tissue samples were frozen on ice and brought to the laboratory. We used aseptic dissection to obtain ~25 mg of tissue from each sample. To avoid contamination, we disinfected and autoclaved the dissection tools after dissecting the samples from each bear and cleaned the tools with 70 % ethanol and 5 % bleach between dissecting the different tissues from the same bear. The 25-mg tissue samples were placed in individual Eppendorf tubes (1.7 ml) and were kept at −20 °C until further analysis.

### DNA extraction of tissue samples

We extracted the DNA from the bear tissue samples using the DNeasy Blood and Tissue extraction kit (QIAGEN) and following the manufacturer’s instructions. We eluted the DNA in 200 μl of AE buffer. We also extracted DNA from the ear tissue samples of four laboratory mice that had been infected experimentally with *B. afzelii* (positive controls) and four laboratory mice that had not been infected with *B. afzelii* (negative controls).

### Quantitative PCR

We used qPCR to detect *Borrelia* spirochetes in the bear tissue samples. We amplified the *flagellin* gene (132 bp) of the *B. burgdorferi* s. l. genospecies complex*.* Details of the primers and probe, qPCR reaction mixture, and thermocycling conditions have been described elsewhere [47, 48]. For amplification we used a LightCycler® 96 (Roche Applied Science, Switzerland). The qPCR plates contained 80 bear tissue samples, the 4 mouse positive DNA extraction controls, the 4 mouse negative DNA extraction controls, 3 negative qPCR controls (pure water), and three standards containing 10^3^, 10^4^, and 10^5^ copies of the *flagellin* gene (three standards on each plate). All samples were run in duplicate using two different qPCR plates.

### Statistical analysis

We used the software program R (version 3.1.2) for the statistical analysis [49]. We calculated the strength of the antibody response against each tick-borne pathogen as the area under the curve of the absorbance versus time plot by using the ‘auc’ function of the R package ‘MESS’ [50]. We refer to this antibody response variable as the optical density. The log-transformed optical densities followed a normal distribution. We therefore analysed this response variable as a linear mixed effects model by using the ‘lme’ function of the R package ‘nlme’ [51]. The log-transformed optical density was modelled as a function of four explanatory variables: study area, age group, year of capture, and bear identity. Study area was a fixed factor with two levels: the northern area and the southern area. Age group was a fixed factor with five levels: yearlings, juveniles, young adults, adults, and old adults. Year of capture was a continuous covariate and was rescaled so that the years 1995 and 2012 corresponded to years 1 and 18, respectively. Bear identity was modelled as a random factor.

We ran 19 candidate models that differed in the structure of the fixed effects, but always with the same random effects structure. The full model contained the three main effects, the three 2-way interactions, and the one 3-way interaction. For the other models, we removed one or more factors and interactions. The corrected Akaike information criterion (AICc) was used to compare models by running the ‘dredge’ function in the R package ‘MuMIn’ [52]. The AICc weight indicated the support for each model. To calculate the support for each explanatory variable, we summed the supports for all the models containing that particular explanatory variable. We used model averaging to calculate a weighted average of the parameter estimates across the set of candidate models. This approach incorporates the uncertainty due to model selection in the calculation of the confidence intervals and provides robust parameter estimates [53].

We tried adding sex as a fourth fixed factor but the models had trouble converging. To test whether the fixed factor sex was important, we repeated the above analyses by replacing the covariate year of capture with the fixed factor sex. All models with the fixed factor sex had lower AICc values than the corresponding models with the fixed factor year of capture. We therefore did not further consider the fixed factor sex in our model selection results.

### Repeatability of the optical density

For the *B. burgdorferi* s. l. ELISA assay, we estimated the repeatability of the optical density for (1) the bear serum samples (two plates) and (2) the controls (14 plates). For the repeatability of the bear serum samples, we used the data from the randomly selected samples of 80 bear sera that were processed twice in two independent ELISA plates. For the repeatability of the controls, we used the data from the positive and negative controls (four *B. afzelii*-infected laboratory mice, four uninfected laboratory mice, and four brown bears from a zoo) that had been used in all 14 ELISA plates.

We used Pearson’s correlation test to determine whether there was a correlation between the optical densities of the *B. burgdorferi* s. l. ELISA assay and the TBEV ELISA assay for the same sample.

## Results

### Repeatability of the optical density of the *Borrelia* ELISA assay

For the *B. burgdorferi* s. l. ELISA, the repeatability of the optical density between the two plates was 0.85 with the controls (F_95_, _96_ = 12.10, *p* < 0.001) and 0.75 without the controls (F_83_, _84_ = 6.96, *p* < 0.001). Thus there was substantial repeatable variation among the bear serum samples and the measurement error was not very large (15 to 25 %). The repeatability of the controls among the 15 plates was 0.99 (F_11_, _168_ = 1071.00, *p* < 0.001).

### Anti-*Borrelia* IgG antibody values of the bear sera

The mean anti-*Borrelia* IgG antibody value of the bear sera was 3.28 times higher than the negative controls (uninfected mice sera), whereas the mean anti-*Borrelia* IgG antibody value of the positive controls (infected mice sera) was 11.76 times higher than the negative controls (Table 1). Thus the antibody values of the bear sera were intermediate between the seronegative and seropositive mice sera. The eight bears with the highest antibody values (range = 110.73 to 130.47 units of optical density) were higher than the least seropositive mice sera. The antibody values of our ELISA assay suggested that brown bears had been exposed to the *Borrelia* pathogen.

**Table 1:** Anti-*Borrelia* IgG values of the Scandinavian brown bears and the negative and positive controls

Serum type	N	Mean	SE	Minimum	Maximum
Wild bears	1172 serum samples (569 individuals)	43.25	0.59	10.17	130.47
Negative controls	56 serum samples (4 individuals)	13.19	0.29	9.10	18.46
Positive controls	56 serum samples (4 individuals)	155.09	2.76	110.60	196.43

The negative controls were uninfected laboratory mice and the positive controls were laboratory mice that had been experimentally infected with *B. afzelii* (Jacquet M, Durand J, Rais O, Voordouw M: Cross-reactive acquired immunity influences transmission success of the Lyme disease pathogen, Borrelia afzelii, submitted). The anti-*Borrelia* IgG response was measured in units of optical density (OD units). The sample size (N), mean optical density, standard error (SE), minimum and maximum values are also shown

### Model selection

In our candidate set of 19 models, the confidence set containing the top four models (1, 2, 3, 4) had a combined support of 96.0 % (Table 2). None of the remaining 15 models had more than 3.0 % of the support (Table 2). The top model had 2.7 times more support than the second-best model (Table 2). There was strong support for the main effects of study area (>99 %), age group (>99 %), and the interaction between study area and age group (>96 %). There was weaker support for the main effect of capture year (>82 %) and the interaction between study area and capture year (>62 %).

**Table 2:** Model selection results of the anti-*Borrelia* IgG response of the Scandinavian brown bears

Rank	Fixed effects structure	df	LL	AICc	Δ AICc	Weight 1	Weight 2
1	OD ~ S + A + Y + S:A + S:Y	14	−442.26	912.53	0.00	0.54	0.54
2	OD ~ S + A + Y + S:A	13	−444.28	914.55	2.02	0.20	0.74
3	OD ~ S + A + S:A	12	−445.36	914.73	2.20	0.18	0.92
4	OD ~ S + A + Y + S:A + S:Y + A:Y + S:A:Y	22	−437.00	917.99	5.46	0.04	0.96
5	OD ~ S + A + Y + S:Y	10	−449.27	918.53	6.00	0.03	0.99
6	OD ~ S + A + Y + S:A + S:Y + A:Y	18	−442.01	920.02	7.49	0.01	1.00
7	OD ~ S + A + Y + S:A + A:Y	17	−444.04	922.09	9.56	0.00	1.00
8	OD ~ S + A + Y	9	−452.70	923.40	10.87	0.00	1.00
9	OD ~ S + A	8	−453.79	923.58	11.05	0.00	1.00
10	OD ~ S + A + Y + S:Y + A:Y	14	−448.95	925.89	13.36	0.00	1.00
11	OD ~ A + Y	8	−455.03	926.06	13.53	0.00	1.00
12	OD ~ A	7	−456.27	926.55	14.02	0.00	1.00
13	OD ~ S + A + Y + A:Y	13	−452.29	930.58	18.05	0.00	1.00
14	OD ~ A + Y + A:Y	12	−454.52	933.04	20.51	0.00	1.00
15	OD ~ S + Y + S:Y	6	−576.20	1164.4	251.87	0.00	1.00
16	OD ~ S + Y	5	−581.93	1173.86	261.33	0.00	1.00
17	OD ~ Y	4	−583.91	1175.81	263.28	0.00	1.00
18	OD ~ S	4	−586.54	1181.07	268.54	0.00	1.00
19	OD ~ 1	3	−588.91	1183.81	271.28	0.00	1.00

The log-transformed optical density (OD) is a measure of the anti-*Borrelia* IgG response and was modelled as a linear mixed effects model. Fixed factors included study area (S), age group (A), and capture year (Y), and the random factor was bear identity. Shown for each model are: the model rank (Rank), the structure of the fixed effects, the degrees of freedom (df), the log-likelihood (LL), the corrected Akaike information criterion (AICc), the difference in AICc value from the top model (Δ AICc), the model weight (Weight 1), and the cumulative weight (Weight 2)

### Age group and study area

**Fig. 2:**
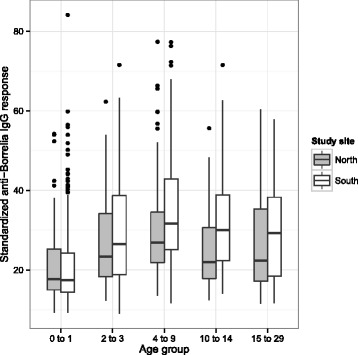
Anti-*Borrelia* IgG response of the Scandinavian brown bears differed among age groups and areas in Sweden. The anti-*Borrelia* IgG antibody response of the bear sera was expressed as a percentage of the mean of the positive controls (mice experimentally infected with *B. afzelii*). Each combination of age group and area contained serum samples from all years of the study (1995 to 2012). The north and south study areas are shown in grey and white, respectively. Shown are the median (black line), the 25th and 75th percentiles (edges of the box), the minimum and maximum values (whiskers), and the outliers (solid circles)

There was substantial variation in the anti-*Borrelia* IgG immune antibody response across age groups (Fig. 2). All other age groups had a higher anti-*Borrelia* IgG antibody response than yearlings (Fig. 2). The immune response in the older age groups was 4.7 to 12.4 % higher than the yearlings, depending on the particular combination of age group and area (Fig. 2). The immune response peaked in the young adult bears (Fig. 2), before declining by 1.4 to 5.0 % in the older age classes, depending on the particular combination of age group and area. The immune response in the south was 0.9, 2.0, and 5.6 % higher than the north for the juveniles, young adults and adults, respectively. In contrast, the immune response in the south was 2.3 and 0.1 % lower than the north for the yearlings and old adults, respectively. Our analysis found that age group and study area were important predictors of the anti-*Borrelia* IgG response in bear sera.

### Capture year and study area

**Fig. 3:**
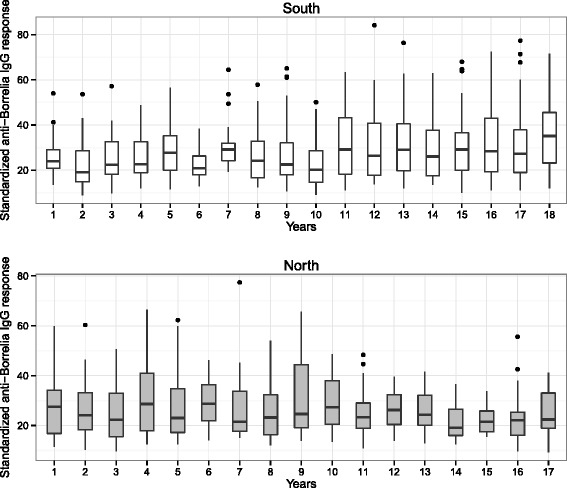
Anti-*Borrelia* IgG response of the Scandinavian brown bears over time differed between areas. The anti-*Borrelia* IgG response in the bear sera is shown for the entire study (year 1 = 1995 and year 18 = 2012). The anti-*Borrelia* IgG antibody response of the bear sera (optical density) was expressed as a percentage of the mean of the positive controls (mice experimentally infected with *B. afzelii)*. Each combination of age group and area contains serum samples from all age classes. The north and south study areas are shown in grey and white, respectively. Shown are the median (black line), the 25th and 75th percentiles (edges of the box), the minimum and maximum values (whiskers), and the outliers (solid circles)

In the southern area, the anti-*Borrelia* IgG response increased slightly over time (Fig. 3). By contrast, in the northern area there was too much variation among years to detect any temporal trend (Fig. 3). For the yearlings in the northern area, the slope of the regression of the anti-*Borrelia* IgG immune response versus time was essentially zero (slope = −0.002 OD units/year, 95 % CL = −0.013 to 0.009 OD units/year; Table 3). For the yearlings in the southern area, the slope was positive and greater than that of the yearlings in the northern area (contrast in slope = 0.011 OD units/year, 95 % CL of the contrast = −0.001 to 0.023 OD units/year) and the lower 95 % confidence limit overlapped zero by a very small amount (Table 3). Our analysis suggested that the anti-*Borrelia* IgG response increased over time, but only in the southern study area.

**Table 3:** Model-averaged parameter estimates of the anti-*Borrelia* IgG response of the Scandinavian brown bears

Parameter	Model-averaged coefficients	Estimate	% Change	SE	Adj SE	z value	*p*	Sig
Intercept^a^	Intercept (yearlings in northern area)	3.421		0.0574	0.0575	59.513	<0.0001	***
Contrast	Juveniles	0.216	6.3 %	0.0613	0.0614	3.524	0.0004	***
Contrast	Young adults	0.350	10.2 %	0.0531	0.0532	6.569	<0.0001	***
Contrast	Adults	0.160	4.7 %	0.0871	0.0872	1.832	0.0669	
Contrast	Old adults	0.237	6.9 %	0.1240	0.1242	1.91	0.0561	
Contrast	Southern area	−0.079	−2.3 %	0.0790	0.0791	0.994	0.3202	
Contrast	Juveniles in southern area	0.113	3.3 %	0.0724	0.0725	1.559	0.1189	
Contrast	Young adults in southern area	0.154	4.5 %	0.0643	0.0645	2.389	0.0169	*
Contrast	Adults in southern area	0.292	8.5 %	0.0997	0.0999	2.92	0.0035	**
Contrast	Old adults in southern area	0.074	2.2 %	0.1552	0.1555	0.477	0.6335	
Slope^b^	Capture year (yearlings in northern area)	−0.002		0.0054	0.0054	0.315	0.7525	
Contrast	Capture year in southern area	0.011	0.3 %	0.0059	0.0059	1.942	0.0521	
Contrast	Capture year in juveniles	0.006	0.2 %	0.0108	0.0108	0.571	0.5681	
Contrast	Capture year in young adults	−0.003	−0.1 %	0.0095	0.0095	0.306	0.7595	
Contrast	Capture year in adults	0.014	0.4 %	0.0147	0.0147	0.95	0.3421	
Contrast	Capture year in old adults	0.024	0.6 %	0.0195	0.0195	1.217	0.2238	
Contrast	Capture year in juveniles in southern area	−0.011	−0.3 %	0.0133	0.0133	0.858	0.3907	
Contrast	Capture year in young adults in southern area	0.010	0.3 %	0.0122	0.0122	0.816	0.4145	
Contrast	Capture year in adults in southern area	−0.025	−0.7 %	0.0172	0.0172	1.43	0.1529	
Contrast	Capture year in old adults in southern area	−0.043	−1.3 %	0.0215	0.0215	2.019	0.0434	*

The parameter estimates for the anti-*Borrelia* IgG response of the brown bears were averaged over the candidate models in Table 2. Anti-*Borrelia* IgG values were measured in units of optical density (OD units) and were modelled as a function of study area, age group, capture year and their interactions. The intercept and slope are defined for the reference group (yearling bears in the northern area). The contrasts refer to the difference in the intercepts (or slopes) between each particular combination of age group and area and the reference group. The percentage change (% Change) expresses each contrast as a percentage of the intercept (3.421 OD units). Also shown are the standard error (SE), the adjusted standard error (Adj SE), the z value, the *p* value (*p*), and the statistical significance (Sig)

^a^Intercept refers to the mean OD for the yearling bears in the northern area

^b^Slope refers to the change in OD per year for the yearling bears in the northern area

Significance codes: ‘***’ = *p* < 0.001; ‘**’ = 0.001 < *p* < 0.010; ‘*’ = 0.010 < *p* < 0.050

### Other interaction terms

Support for the interaction between capture year and age group was weak. The two models that contained this interaction had a combined support of 5 % (Table 2). Thus there was little evidence that the interaction between capture year and age group influenced variation in the anti-*Borrelia* IgG response of the bears. Support for the three-way interaction between area, age class, and capture year was also weak. The one model that contained this three-way interaction had a support of 4 % (Table 2).

### Repeatability of the optical density of the TBEV ELISA assay

Difference in background absorbance between the two plates overwhelmed the variance in absorbance among the samples. The repeatability between the two plates was therefore calculated after standardizing the optical density values to z-scores for each plate. For the TBEV ELISA assay, the repeatability of the standardized optical density between the two plates was 0.80 with the controls (F_95_, _96_ = 9.24, *p* < 0.001) and 0.58 without the controls (F_76_, _77_ = 3.80, *p* < 0.001). Thus there was substantial repeatable variation among the bear serum samples and the measurement error was moderate (20 to 42 %). The repeatability of the controls among the 15 plates was 0.97 (F_10_, _165_ = 462.80, *p* < 0.001).

### Anti-TBEV IgG antibody values of the bear sera

The mean anti-TBEV IgG antibody value of the bear sera was 2.31 times lower than the negative controls (uninfected goat sera) and 16.37 times lower than the positive controls (infected goat sera) (Table 4). The twelve bears with the highest antibody values (range 23.04 to 39.77 units of optical density) were higher than the mean antibody value of the seronegative goat sera (22.75 units of optical density). The ELISA results suggested very weak exposure of brown bears to TBEV and we therefore did not further analyse these data.

**Table 4:** Anti-TBEV IgG values of the Scandinavian brown bears and the negative and positive controls

Serum type	N	Mean	SE	Minimum	Maximum
Wild bears	1172 serum samples (569 individuals)	9.86	0.11	1.93	39.77
Negative controls	60 serum samples (4 individuals)	22.75	0.99	7.59	47.53
Positive controls	60 serum samples (4 individuals)	161.37	2.85	82.84	185.83

The negative controls were uninfected goats and the positive controls were goats that tested positive for TBEV [46]. The anti-TBEV IgG response is measured in units of optical density (OD units). The sample size (N), mean optical density, standard error (SE), minimum and maximum values are also shown

### Correlation in optical density between *Borrelia* and TBEV assays

There was a significant, positive correlation between the anti-*Borrelia* IgG immune response and the anti-TBEV IgG immune response across the bear serum samples (Pearson’s correlation coefficient = 0.156, N = 1141, *p* < 0.001). For the twelve combinations of age group and area, the correlation between the two immune responses was always positive and in some cases statistically significant (Table 5).

**Table 5:** Correlation in optical density for *Borrelia* and TBEV ELISA assays in the Scandinavian brown bears

Area	Age group	N	r	*p*
North	Yearlings (0–1)	124	0.066	0.4642
North	Juveniles (2–3)	58	0.110	0.4101
North	Young (4–5)	56	0.188	0.1661
North	Middle (6–9)	86	0.409	<0.0001
North	Old (10–14)	40	0.126	0.4398
North	Older (15–29)	20	0.188	0.4265
South	Yearlings (0–1)	245	0.130	0.0414
South	Juveniles (2–3)	130	0.198	0.0240
South	Young (4–5)	114	0.175	0.0631
South	Middle (6–9)	129	0.069	0.4340
South	Old (10–14)	86	0.171	0.1164
South	Older (15–29)	53	0.341	0.0124

Pearson’s correlation coefficient for the optical density between the *Borrelia* and TBEV assays is positive for all 12 combinations of age group and area for brown bears in Sweden. The area, age group, sample size (N), Pearson’s correlation coefficient (r) and *p*-value (*p*) are shown

### Analysis of the bear tissue samples using qPCR

The qPCR worked well, as 90 % of the positive controls tested positive for *Borrelia* spirochetes (9 positive/10 total; 6/6 standards and 3/4 ear tissue samples from experimentally infected mice tested positive for *B. afzelii*) and all negative controls tested negative. None of the bear tissue samples tested positive for *B. burgdorferi* s. l. pathogens.

## Discussion

The anti-*Borrelia* immune response was higher in the southern bears than the northern bears for all age groups (except yearlings and old adults). This pattern is consistent with the geographic distribution of *I. ricinus*, which is more common in southern Scandinavia where the climate is warmer [9, 11]. The bears in the southern area were captured primarily in the counties of Dalarna and Gävleborg. In these two counties, populations of *I. ricinus* increased substantially from the early 1990s to 2008 [10]. The bears in the northern area were captured primarily in the northwestern corner of Norrbotten County. In 1990, there were reports of *I. ricinus* in the coastal area of Norrbotten County bordering the Gulf of Bothnia [10]. In 2008, *I. ricinus* was reported in central Norbotten County, and this focus overlapped with some of the sampling locations of the bears. In summary, the range maps of *I. ricinus* in the study by Jaenson *et al.* [10] suggest that the bears in the southern area are more likely to encounter ticks than the bears in the northern area. Furthermore, stable, high-density populations of ticks are more favourable for the introduction and maintenance of tick-borne pathogens [54]. The higher anti-*Borrelia* IgG response in the southern bears was therefore consistent with the expected higher abundance of ticks in southern Scandinavia. We also found that the anti-*Borrelia* IgG immune response in the bear population was much stronger than the anti-TBEV IgG immune response. This difference was not surprising because *Borrelia* pathogens are much more common than TBEV in populations of *I. ricinus* ticks [20].

The mean anti-*Borrelia* IgG immune response in the bears increased over time, but only in the southern area. This observation is consistent with the literature documenting that the prevalence of ticks and tick-borne diseases (e.g. LB and TBE) has increased in Scandinavia over the last three decades [1–7, 9, 14]. Numerous authors have suggested that climate change is causing this increased burden of tick-borne diseases [11, 55, 56], whereas others have argued against this view [3, 19]. The temporal increase in anti-*Borrelia* IgG levels in the southern bears over the last 18 years is consistent with an increased abundance of *Borrelia*-infected ticks in southern Scandinavia. An alternative explanation for the time-dependent increase in the anti-*Borrelia* immune response is time-dependent, cumulative damage to the bear serum samples. However, if this explanation was true, we should have observed the same time-dependent increase in the anti-*Borrelia* IgG response in the northern area.

The older bears generally had a stronger anti-*Borrelia* IgG immune response than younger bears. Age-related increases in seropositive status are commonly observed, because the probability of encountering a pathogen increases throughout an individual’s lifetime [57, 58]. Similar patterns have been observed in wild mice, where adult individuals typically have higher anti-*Borrelia* IgG levels than sexually immature individuals [59, 60]. The observation that the anti-*Borrelia* IgG immune response increased over the first three age classes may be explained by the development of the immune system. Mammals build up their immune system by encountering a wide variety of pathogens during development [61, 62]*.* The decrease in the immune response of the older bears suggests immuno-senescence. Such age-related declines in the acquired immune response also have been observed in human populations [63, 64].

*Ixodes* ticks are capable of feeding on bears [26, 29, 65–68]. Numerous studies in North America have collected *I. scapularis* and other tick species from the American black bear (*Ursus americanus*) [26, 28, 29, 65, 66]. Serological studies further suggest that *U. americanus* is frequently exposed to *Borrelia* pathogens [69, 70]. A molecular screening of brown bears in Slovakia found that 24.3 % (18/74) of the animals tested were positive for the tick-borne pathogen *Anaplasma phagocytophilum* [67]. A serological survey of brown bears in Slovakia found that 65.2 % (15/23) of the animals were seropositive for *A. phagocytophilum* [68]. As *I. ricinus* is the principal vector of *A. phagocytophilum*, these two studies provide indirect evidence that *I. ricinus* ticks are capable of feeding on brown bears [67, 68].

Mammalian hosts differ substantially in their ability to maintain systemic infections with *Borrelia* pathogens [71]. Competent hosts, such as rodents, can maintain long-lived, chronic infections in their tissues [72, 73]. Incompetent hosts, such as deer, do not develop systemic infections [74–76], but such hosts can still develop a strong antibody response to *Borrelia* pathogens [77, 78]. Whether or not bears are competent hosts for *Borrelia* pathogens is currently unknown. A study on Lyme disease in the American black bear isolated spirochetes from blood and kidney samples, but no PCR was conducted to confirm pathogen identity [26]. In our study, none of the tissue samples from the 16 bears tested positive for *B. burgdorferi* s. l. DNA. Consistent with our results, previous studies also have shown that bears develop antibodies against *Borrelia* pathogens [69, 70]. It is possible that bears, like deer, may act as sentinel hosts for *Borrelia* pathogens without developing a systemic infection.

Cross-immunity is a potentially confounding factor in any serological survey. Antibodies developed against other pathogens could be cross-reactive with the antigens used in our assays. For example, antibodies developed against *Treponema* sp*.* in humans can cross-react with the antigens of *Borrelia burgdorferi* s. l. pathogens [79, 80]. Thus we cannot exclude the possibility that unknown microbial pathogens of the brown bear may have contributed to the background absorbance observed in the serum samples of this study. Future studies should use immunoblotting to further confirm the specificity of the anti-*Borrelia* IgG antibody response in brown bears and other wild animal populations.

## Conclusions

Our long-term serological study of the Scandinavian brown bear provides evidence consistent with the observation that ticks and tick-borne pathogens are expanding their abundance and prevalence, respectively, in northern Europe. Bears in the southern area, where *I. ricinus* ticks have been reported, had higher values of anti-*Borrelia* IgG antibodies than bears at the northern area, where *I. ricinus* ticks are believed to be less common. Over the 18 years of the study, the value of anti-*Borrelia* IgG antibodies increased in the southern area, but not the northern area. Our study suggests that long-term serological monitoring of large mammals can provide insight into changes in the distribution of ticks and tick-borne diseases, and perhaps the reasons for these changes.

## Abbreviations

AICc: Akaike information criterion
LB: Lyme borreliosis
TBE: tick-borne encephalitis
TBEV: tick-borne encephalitis virus
